# The Use of Terrestrial and Maritime Autonomous Vehicles in Nonintrusive Object Inspection

**DOI:** 10.3390/s22207914

**Published:** 2022-10-18

**Authors:** Dmytro Mamchur, Janis Peksa, Antons Kolodinskis, Maksims Zigunovs

**Affiliations:** 1 Information Technologies Department, Turiba University, Graudu Street 68, LV-1058 Riga, Latvia; 2 Computer Engineering and Electronics Department, Kremenchuk Mykhailo Ostrohradskyi National University, Pershotravneva 20, 39600 Kremenchuk, Ukraine; 3 Institute of Information Technology, Riga Technical University, Kalku Street 1, LV-1658 Riga, Latvia

**Keywords:** self-driving vehicle, artificial intelligence, classification, nonintrusive inspection

## Abstract

Traditional nonintrusive object inspection methods are complex or extremely expensive to apply in certain cases, such as inspection of enormous objects, underwater or maritime inspection, an unobtrusive inspection of a crowded place, etc. With the latest advances in robotics, autonomous self-driving vehicles could be applied for this task. The present study is devoted to a review of the existing and novel technologies and methods of using autonomous self-driving vehicles for nonintrusive object inspection. Both terrestrial and maritime self-driving vehicles, their typical construction, sets of sensors, and software algorithms used for implementing self-driving motion were analyzed. The standard types of sensors used for nonintrusive object inspection in security checks at the control points, which could be successfully implemented at self-driving vehicles, along with typical areas of implementation of such vehicles, were reviewed, analyzed, and classified.

## 1. Introduction

The task of nonintrusive object inspection has become crucial nowadays due to increased goods flow and people flow in recent decades. With this increase, the number of potential threats increases as well, as in crowded places among a number of people, parcels, and baggage it is easier to hide dangerous or illegal items that smugglers could use, as well as terrorists and other criminals. On the other hand, overcontrolling these flows is highly undesirable, creating obstacles and delays in free traveling and delivery. Thus, there is an urgent need to develop nonintrusive unobtrusive methods for passive surveillance to identify potential threats in crowded places or at public checkpoints. Besides traditional nonintrusive methods used at stationary checkpoints, as described in [1], with the advance in robotics, it has become possible to extend inspection systems’ equipment to mobile self-driven platforms. Such an approach is beneficial for maritime inspection and might be used for on-land purposes. Nonintrusive object inspection using self-driving vehicles could be active and passive [1,2,3]. During the functional assessment, different types of control points are established, where people and their belongings should pass through security gates or could be inspected with traditional equipment, such as a manual metal detector, or be searched with the help of specially trained animals (dogs, rats) [4,5,6]. During a passive inspection, security should be ensured without direct interaction with the object of the search, even without informing them about the searching process [7,8,9]. With this aim, different object and behavior recognition techniques are employed, typically based on processing the images received from surveillance video cameras installed in public places [10,11,12,13,14].

One relatively novel approach in the last few decades is social media analysis [15,16,17,18]. The idea behind this approach is social media profile analysis to identify suspicious persons in the early stages of their appearances in public place [17]. These methods could be automatic, using artificial intelligence and image recognition algorithms to identify specific persons, followed by a search of their social media profiles with activity analysis. These methods are relatively novel and have limited implementation nowadays due to nonsatisfiable search and analysis quality. However, they are constantly improving with the increasing knowledge base and advances in sensors and methods used for this type of analysis [19,20,21,22,23]. 

In the case of traditional nonintrusive object inspection, in some instances, it is impossible to use fixed control points. Thus, mobile and self-moving vehicles might help [24,25,26,27,28]. As an example, such inspection could be provided for underwater object surveillance during border control, when it is not easy to give a traditional type of nonintrusive inspection with the fixed control point over large vehicles, such as cruise liners, or at a vast area, such as port or a gulf. In this case, smaller remotely controlled or self-driven vehicles equipped with a set of sensors, transducers, and other technologies for nonintrusive control are in use to provide security inspection activities [29,30,31,32]. Such vehicles have proven to be effective for underwater inspection, but they still need to be improved with more advanced sensors and signal processing algorithms [33]. Similarly, autonomous self-moving on-land vehicles could be used for nonintrusive inspection in crowded places to increase public safety with early suspicious object detection. To make reviewing more efficient, the process also should be automated. One of the possible solutions is to employ self-moving vehicles carrying inspection equipment so that they could autonomously inspect massive objects, crowds, or object flows with the help of nonintrusive control; analyze information; and send alarms to security officers in case of suspicious situation being detected [34]. 

## 2. Typical Structure of Self-Moving Vehicles

As a carrier for nonintrusive object inspection equipment, self-driven autonomous vehicles could be used. Typically, these vehicles contain a set of sensors to analyze an environment detecting obstacles on their preplanned route, and a processing unit to control the vehicle’s movement and recalculate the route depending on the presence of obstacles [35,36]. 

**Figure 1:**
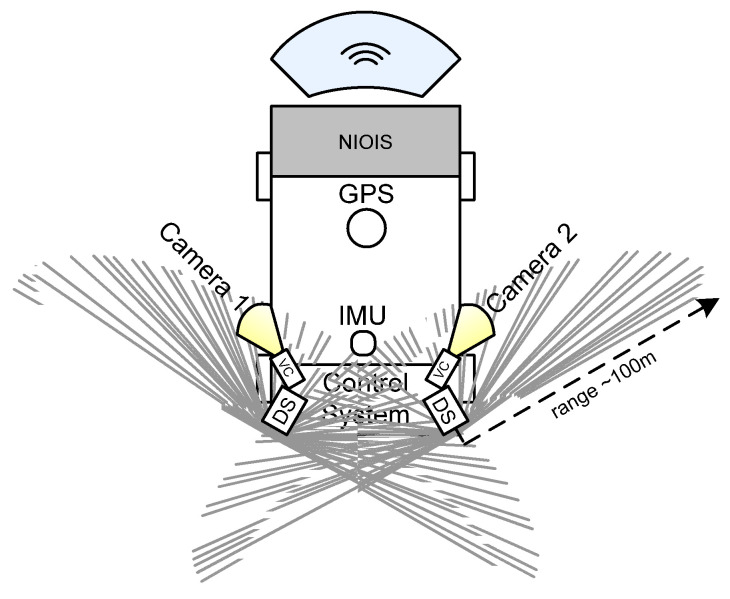
Typical structure of terrestrial self-driving vehicles equipped with nonintrusive object inspection system. Top view.

The typical structure of on-land self-driving vehicles that could be used for nonintrusive object inspection is shown in Figure 1. Maritime vehicles have a similar structure, except for the drive system and set of sensors adapted for maritime use. 

Such vehicles are typically equipped with distance sensors (DS), used to detect distance to the nearest obstacle, and video cameras (VC), to provide image analysis for obstacle detection and object classification. A GPS detects the current vehicle’s location, inertial measurement units (IMU) collect data from gyroscopes, accelerometers, and magnetometers about vehicle motion, and a control system processes all data and implements motion control algorithms. For nonintrusive inspection purposes, the vehicle is additionally equipped with a nonintrusive object inspection system (NIOIS), which could include different types of sensors and data-processing software depending on the environment under surveillance and inspection tasks, such as portable X-ray sensors, infrared cameras, acoustic sensors, odor sensors, etc. [37]. The following chapter reviews sensors used for self-driving motion control.

## 3. Sensors Used for Self-Driving Vehicles

Interference avoidance is essential in developing motion control systems for self-driven vehicles. This task is typically solved using different types of sensors and control algorithms executed on microcontrollers or microcomputers. These special-purpose computing devices typically contain pins and ports to accept sensor information via standard industrial wired or wireless data-transfer protocols. 

In general, the most common method for obstacle detection uses either or both of two types of sensors: active sensors, such as various types of laser and ultrasonic distance sensors; and passive sensors, such as video cameras [38,39,40,41]. All sensors vary by their effective range, possibility to detect close and remote objects, operational speed, viewing angle, and overall price. The typical sensor types used for obstacle detection and their main features are presented in Table 1.

As can be seen from Table 1, different sensors are used with different purposes for object detection. Thus, to detect close objects, ultrasonic sensors are the best choice. However, their effective distance and viewing angle are poor, although the price is very attractive. On the other hand, LIDAR and RADAR sensors have 360° viewing angle, good effective distance, but quite a high price. Thus, the choice of sensor type to implement the interference avoidance algorithm should be made carefully depending on the purpose of the self-driving vehicle. For example, for road vehicles—which themselves cost thousands of dollars, and for which a typical task consists of detecting relatively remote objects, within tens or hundreds meters distance—it is reasonable to implement RADAR and LIDAR sensors. However, for small self-driving drones, where it is quite important to create relatively cheap devices that also should move with relatively slow speed, it is reasonable to consider using a set of ultrasonic sensors. Finally, if the aim is to develop a relatively cheap device that could align with object detection and perform an object recognition algorithm based on its image, the use of a video camera might be considered. In tasks of nonintrusive object inspection with the use of autonomous vehicles, a 360° viewing angle and speed detection are not the most-needed options, while it is important to detect close objects and image recognition technology might be in use. Thus, it is reasonable to use a combination of an ultrasonic sensor and a video camera for this purpose.

### 3.1. Obstacle Detection with the Video Camera

This method typically employs one or two video cameras to detect obstacles based on image-processing techniques. 

In the case of a single camera, the following algorithms are used:

- Algorithms based on known object recognition. They aim to recognize previously known object parameters and evaluate the distance to these objects based on known object dimensions. These algorithms are easy to implement and they are able to effectively recognize previously known objects, but are useless under any uncertainties, e.g., detecting obstacles that were not previously learned;
- Motion-based algorithms. They analyze the sequence of images and compute each pixel offset. Based on the system motion data, it is possible to detect obstacles that appear on a vehicle way without previous information on the type or shape of these obstacles. In most cases, an optical flux algorithm is used to implement this method.

In the case of stereo vision, i.e., combining information from two cameras, the following algorithms are used:

- The stereo comparison method, based on searching for common patterns in two images, computing differences in the binocular discrepancy maps, and estimating the distance to the obstacle based on the horizontal displacement;
- The method of homographic transformation, which aims to transform the image angle from one camera to the image angle of another camera, assuming any significant differences between straight and altered images as an obstacle.

Typically, a combination of the above-mentioned methods is practically used, providing sufficient information about the environment and obstacles. This information could be used in complex route-planning algorithms for self-driving vehicles. Among the significant disadvantages of interference detection methods with video cameras are their high cost and high computational complexity of the models used, which require the use of neural networks and high-performance computing devices, which, in turn, also have a high cost and energy consumption, which are especially important in the development of mobile devices [42,43].

### 3.2. Interference Detection Using Active Sensors

Active sensors use reflected signal analysis to compute the distance to an obstacle. This group of sensors includes LIDAR (light detection and ranging), RADAR (radio detection and ranging), ultrasonic sensors, infrared sensors, and others. The first three are the most popular in this group:

- LIDAR uses laser radiation to calculate the distance to the target;
- RADAR uses radio waves to calculate the angle, type, distance, and speed of the obstacle;
- Ultrasonic sensors use high-frequency sonic radiation to analyze the time of reflected signal detection to determine the distance to the object.

Compared to video cameras, the advantages of these methods are the possibility to distinguish objects and obstacles under different lighting conditions, higher operational range, specificity, and accuracy of obstacle detection. In addition, less computational capacity is required to calculate obstacle parameters, as these sensors’ operational principle does not require complex computation for image processing. Each method has its pros and cons, as in Table 1 [44,45,46,47,48,49,50,51,52].

### 3.3. Rationale for the Obstacle Sensor Choice

Data from Table 1 show that using LIDAR sensors for small autonomous vehicles and small robots is not economically reasonable. Thus, most commercial and industrial applications use RADAR or video cameras for obstacle detection. However, even these sensors could be a bit expensive for simple applications requiring cheap solutions, such as nonintrusive surveillance during small public events. In such cases, cheap infrared and ultrasonic sensors are in use. A detailed comparison of the effectiveness of different obstacle detectors is given in [53].

Based on the provided analysis, it was concluded that for nonintrusive and unobtrusive object inspection with the implementation of self-driving vehicles, in most cases RADAR or a video camera should be implemented as the primary sensor and an ultrasonic sensor as an additional. 

Moreover, it should be mentioned that ultrasonic signal processing is another challenge, due to nontrivial data-processing and control algorithms.

### 3.4. Obstacle Detection Algorithms

The motion of a self-driving vehicle in a changing environment is possible only when the vehicle can adapt its behavior following the changing information about this environment. In most cases, based on the preliminary description of the environment, the route-planning module generates possible paths to a given destination. Using data from sensors in real time, the self-driving algorithm should adjust the vehicle’s motion to avoid collisions via recalculating basic path parameters. The use of ultrasonic distance sensors as the main obstacle detectors is quite problematic in this case, because the raw distance data do not provide information on obstacle location. Thus, complete information on obstacles could only be obtained via examining an obstacle from different angles. As an on-duty self-driving inspection vehicle performs specific tasks, it is impossible to spend time collecting information from each possible angle. Instead, the system should obtain the maximum possible information from the data collected during the vehicle’s movements. The possible solution is collecting and merging data from previous measurements and creating a continuous environment map based on these measurements. However, such an approach, with the use of environment simulation using graphical primitives (lines, polygons, circles, etc.), requires high-resolution information and a considerable data preprocessing capacity. Even after meeting all these requirements, the control system could provide false results due to information noise, errors during data collection, sensor faults, etc.

Alternatively, an environment map could be created based on different types of fullness matrices. There are two types of fullness matrices: raster matrix (fixed-cell matrix) and adaptive cell size matrix. Each of these types has its pros and cons. Using a raster matrix also requires using a large but fixed memory volume. The use of an adaptive cell size algorithm reduces the memory capacity needed in cases of vast obstacles or huge unfilled environment areas. However, it requires a lot of mighty computational power, along with frequent data updates, which also harden the use of probabilistic algorithms.

Similar approaches are used with other obstacle detection sensors, but they vary in distance range for sensor effect, computational capacity, and sensor price, as it was mentioned previously.

## 4. Route Planning Algorithms for Self-Driving Vehicles

Another essential task for self-driving vehicles used for nonintrusive object inspection is an implementation of route-planning and motion control algorithms to cover the whole search area detecting obstacles on their way either to inspect or avoid them, followed by preplanned route recalculation. Route planning for self-driving vehicles is searching for a geometrical interpretation of the vehicle route from starting position to the target using a typically incomplete obstacle map to avoid collision with these obstacles [54]. 

Depending on the vehicle type, environment, and movement control algorithm, different assumptions might be applied to build an environment simulation model and make the path-searching process more efficient. 

Currently, several different algorithms are developed for obstacle avoidance and route planning, starting with the simple vehicle stop when an obstacle is detected and finishing with the vehicle adaptive behavior change depending on the type, dimensions, and other features of the detected block. These algorithms differ in the data amount needed to process, number of sensors, operational speed, spatial complexity, efficiency, and control strategy [55].

The most typical algorithms used in self-driving vehicle control systems could be divided into the following groups:

- Bug algorithms;
- Naïve, or the simplest algorithms;
- Algorithms with the use of distance sensors;
- Potential field-based algorithms;
- Graph-based algorithms;
- Formal algorithms (Dijkstra’s algorithm, Floyd–Warschall algorithm);
- Heuristic algorithms (e.g., width search);
- Hybrid search algorithms (for example, A*, D*).

### 4.1. Bug Algorithms—Bug-1 Algorithm

Bug algorithms implement the most straightforward naïve approach aimed to move directly to the target until encountering an obstacle. In case of collision, the shape of a block is calculated, followed by a recomputation of the path to the target. These algorithms do not save previously collected data and do not use once-created environment maps. Instead, they use only directly measured data [56,57]. The first algorithms from this class were proposed in 1986 by V. Lumelskiy and O. Stepanov and were called Bug-1 and Bug-2. The difference between these algorithms is the condition for stopping the motion alongside the obstacle and continuing movement to the target. 

**Figure 2:**
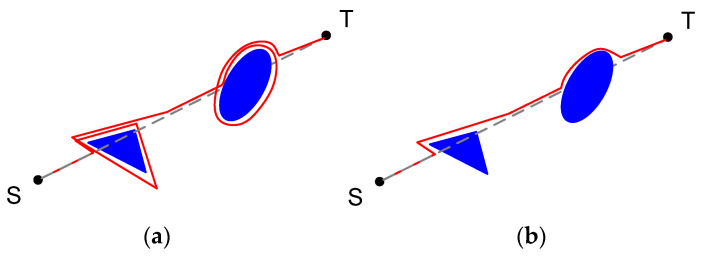
Bug-1 (**a**) and Bug-2 (**b**) algorithms comparison.

In the Bug-1 algorithm, in the case of obstacle detection, a self-driving vehicle keeps its motion around an obstacle starting from the collision point, determining its shape. After this, vehicle calculates the obstacle contour point closest to the target, called the “starting point”. Then, the vehicle moves alongside the obstacle contour to the “starting point”. Reaching this point, the vehicle moves to the target with a newly recalculated route. Such an algorithm is hugely inefficient, but it guarantees to obtain any target point if possible [58,59]. An example of the movement with the Bug-1 algorithm is presented in Figure 2. 

To increase Bug-1 efficiency, it was modernized to Bug-2, where a self-moving vehicle stops its motion around an obstacle once it crosses an M-line or a course-line—a conditional line between initial vehicle position and a target. A comparison of the Bug-1 and Bug-2 algorithms is presented in Figure 2.

The simplicity of these algorithms has several significant drawbacks. First of all, vehicle paths will not be optimal. Secondly, these algorithms do not consider the vehicle’s mechanics [60]. Mainly, such a type of movement is impossible for the car. 

**Figure 3:**
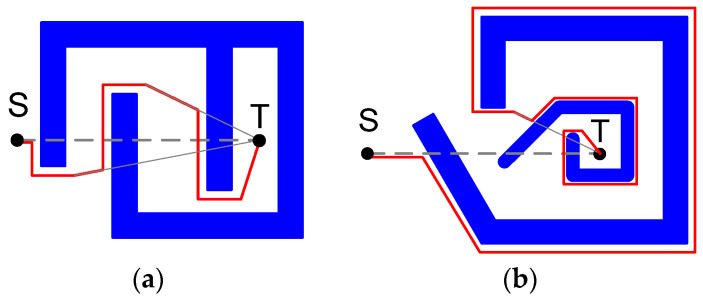
Improvement (**a**) and deterioration (**b**) of the vehicle movement based on the DistBug algorithm.

To solve the first problem, in 1997 I. Kammon and E. Rivlin proposed the DistBug algorithm. Differently to Bug-2, the “starting point” considers the point with the distance to the target less than the distance from the next obstacle point. In addition, in contrast to the Bug-1 and Bug-2 algorithms, the direction of motion alongside the obstacle is calculated depending on the angle with which the vehicle approaches the barrier. Although these modifications significantly reduce the distance to be covered in most cases, this algorithm could lead to opposite results in several instances. An example of both cases is given in Figure 3 [61].

### 4.2. Bug Algorithms with the Distance Sensor

**Figure 4:**
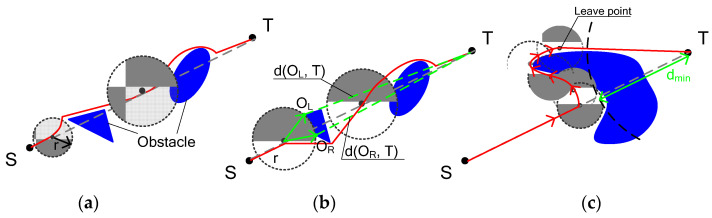
VisBug (**a**) and TangentBug (**b**,**c**) algorithms.

All the algorithms listed above are based on the assumption that the self-moving vehicle can detect the presence of an obstacle only at a close distance to it. However, most modern sensors used in robotics can detect obstacles at a much longer range. Based on these assumptions, Lumelsky and Skwis proposed the VisBug-21 [62] and VisBug-22 [63] modifications of Bug algorithms in 1988 and 1990, respectively. These algorithms are based on Bug-2, i.e., the vehicle follows the “M-line” but significantly reduces the trajectory. Figure 4a shows an example of the algorithm.

Another more successful approach was the TangentBug algorithm, developed by I. Kamon, E. Rivlin, and E. Rimon in 1997 [64] based on the DistBug algorithm. This algorithm is shown in Figure 4b,c. LTG states for ‘local tangent graph’, which is constructed within the distance sensor range. Next, the algorithm searches for obstacle avoidance point Oi, which minimizes the expected sum of distances d(x, O_i_) + d(O_i_, T), where x is the vehicle’s current position and T is the target point. Figure 4c shows when the initially optimal path, supplied with new data, became much longer. In this case, the algorithm saved the d(O_i_, T) value estimation, then continued its move along the obstacle until the distance to the target became shorter than this estimate. After that, the vehicle left the obstacle contour trajectory and continued its move to the target.

**Figure 5:**
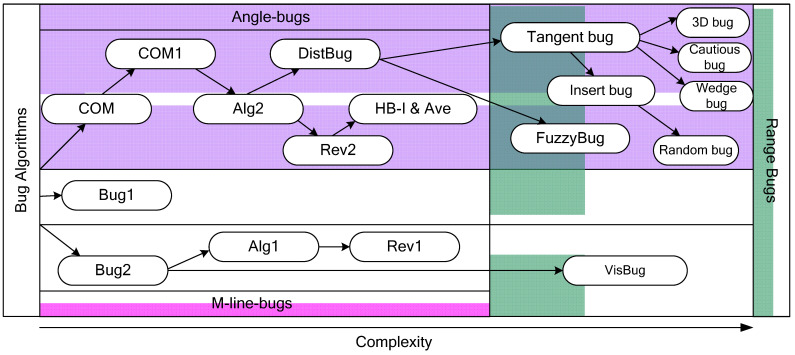
Bug-type algorithms classification.

This algorithm proved to be the most efficient among the Bug family algorithms, and many modifications were created based on it. In particular, the WedgeBug algorithm considers the distance sensor’s limited viewing angle, and the InsertBug algorithm thinks about the safety of the vehicle’s move radius around the obstacle. The general classification of the Bug-type algorithms is shown in Figure 5 [58].

The basic principles of Bug algorithms, as well as Bug1, Bug2 and COM algorithms were presented by Lumelsky and Stepanov in [59,60], and later developed by Kamon and Rivlin in [61]. Insert bug and VisBug were proposed by Lumelsky and Skewis in [62] and developed in [63]. DistBug was proposed by Kamon, Rivlin and Rimon in [64]. Sankaranarayanan and Vidyasagar proposed Alg1 and Alg2 in [65]. Rev1 and Rev2 algorithms were described by Noborio, Maeda and Urakawa in [66]. Horiuchi and Noborio proposed HB-I & Ave algorithm in [67]. Lee, Adams and yeol Ryoo developed FuzzyBug algorithm and presented it in [68]. Finally, Kamon, Rimon and Rivlin proposed Tangent bug algorithm in [69].

### 4.3. Potential Field Algorithms

**Figure 6:**
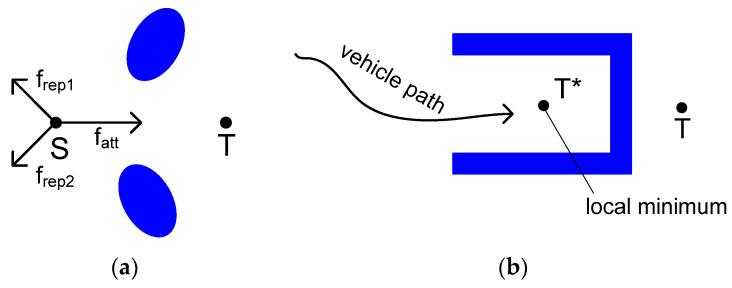
Examples of obstacles moving according to potential field algorithms and stopping before reaching the target point: sum of repulsive vectors balances attraction vector and vehicle stops (**a**); obstacle geometry forces move to local minimum and stop at local target point T* (**b**).

O. Khatib proposed a navigation algorithm based on the use of the artificial potential field. According to this concept, a self-driving vehicle is considered a particle moving in a possible area generated by a destination point and obstacles. The destination point creates the potential for attraction, while blocks generate the potential for repulsion. The vehicle moves in this field under the influence of a force that attracts it to the destination point while repulsing it from obstacles on the route [70]. This potential field is called the vector force field (VFF). The possible area could be constructed both from a previously known obstacles map and based on sensor data obtained during the movement. In the second case, if the information about the field has been changed, a recalculation of the forces influencing the area should be made. This approach has some limitations that make it hardly usable without additional modifications. For example, Figure 6 shows the configuration of obstacles where a self-driving vehicle never reaches its destination.

**Figure 7:**
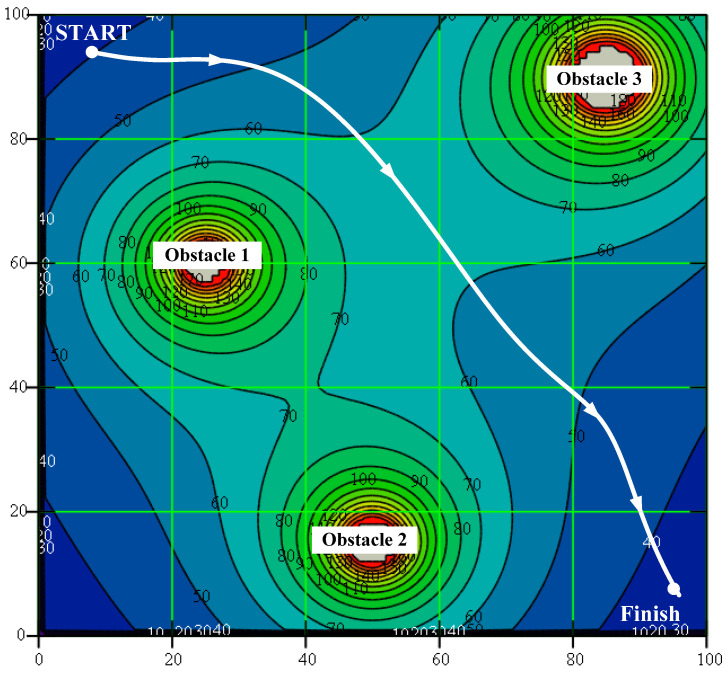
Graphical representation of a potential field.

To avoid the situations shown in Figure 6b, it was proposed to construct a potential field as the sum of two components: the target field, i.e., the direction of movement to the target point; and the barrier field, consisting of repulsive forces. The sum of these components is the navigation field. Such a field can be represented as a slope with mountains, where the potential gradient equals the field inclination. An example of such field representation is shown in Figure 7 [71].

The general idea of the methods is to move along the vector lines of the vector field, the potential function of which reflects the configuration of obstacles and their shape, and also the target point. This approach is suitable both in two-dimensional and three-dimensional environments. Among the potential field methods, the most popular one is the artificial potential field (APF). This algorithm is simple, with low complexity and high implementation efficiency [71]. The vector field is divided into two components: the target point, represented by an attractive vector field; and obstacles, represented by a repulsive vector field. The addition of these two vector fields along the vehicle’s trajectory allows one to solve two tasks: moving to a given target point and avoiding obstacles. The technology of path planning using the artificial potential method is simple, and thus allows one to control the movement in real time. However, the method has a significant drawback: the existence of local minima is possible. A number of approaches have been proposed to eliminate this shortcoming, but there is still no completely satisfactory solution, and these approaches could be used only in certain situations under specified conditions. The method also has an uncertainty associated with the choice of the function coefficients for construction the potential functions. These two factors limit the wide use of the artificial potential method for the solution of practical problems. For example, these algorithms do not allow one to avoid unsolvable situations similar to presented in Figure 6a. 

Various approaches were used to improve this type of algorithm, including the implementation of neural networks, high-level control algorithms, and advanced algorithms where the potential field is calculated by taking into account not only the absolute position of the self-driving vehicle and obstacles, but also the vehicle position relative to the obstacle and ignoring the blocks behind the vehicle. A significant disadvantage of these algorithms is that calculating the potential field requires information about most of the obstacle’s positions within the area, albeit inaccurately. This could lead to erroneous results in a significant incompleteness of the information, and in some cases, may prevent the vehicle from reaching the target point. Another disadvantage is the representation of the vehicle as a particle that does not consider its kinematic parameters (for example, the vehicle cannot make a turn at a significant angle without passing a certain distance).

### 4.4. Algorithms on Graphs

Graph-based algorithms assume dividing space into cells, considered nodes, and creating a graph by connecting adjacent cells with edges of different weights. The boundaries that reach nodes with the obstacles usually indicate infinitely immense importance, which allows one to implement any search algorithm for the graph. The use of standard algorithms such as the Dijkstra or Floyd–Worschel algorithm does not consider the desired motion or other preknown environment parameters, leading to a large number of nodes that need to be processed. For example, the Dijkstra algorithm searches for a destination evenly in all directions, and the Floyd–Worschel algorithm handles all available edges, leading to the algorithm’s quadratic complexity. A heuristic breath-first search algorithm can be used in a predefined heuristic (the simplest case is the distance to the target). The result of such an algorithm will be a vehicle route similar to Bug-type algorithms.

### 4.5. Hybrid Search Algorithms

The idea of the hybrid search algorithm A* is to apply the existing heuristics to the Dijkstra algorithm. Algorithm A* uses heuristics to determine the nodes that should be analyzed first. This would help reduce the Dijkstra algorithm’s running time by reducing the number of nodes under analysis. This preserves the feature of the Dijkstra algorithm concerning the guaranteed pathfinding, and if the heuristic is admissible, the finding of the shortest path is also guaranteed. The most popular are:

(1) Dijkstra’s algorithm;
(2) Width search algorithm;
(3) Algorithm A*.

All the above-listed algorithms can work only with an a priori (preliminary) created environment map and must be restarted once the map changes. To improve the efficiency of these algorithms in dynamically changing environment, two groups of improvements were developed for the A* algorithm, which allow one to update the previously calculated path in case new information appeared:

- D* algorithms: D * and Focused D *;
- LPA* algorithms: LPA* and D* Lite algorithms.

Both options implement the A* algorithm, saving all its parameters. If the information about previously known parts of the environment changes and it is not updated, such preservation causes additional challenges. A significant disadvantage of these algorithms is their spatial complexity, b^2^, where b is the number of nodes or cells of the obstacle’s matrix. Another downside is the necessity to process a significant number of cells each time the data are updated.

### 4.6. Rationale for the Choice of Algorithm

The pros and cons of the reviewed algorithms are summarized in Table 2.

Based on the provided analysis, it could be concluded that algorithms based on the potential field have several disadvantages:

- They require information on the existence of all obstacles, even those that are beyond the scope of the sensors;
- They have great computational complexity, as they provide a complete recalculation of the potential field with each new measurement;
- They do not guarantee the achievement of the goal with specific configurations of obstacles or incomplete initial data;
- They do not take into account the kinematics of the self-driving vehicle.

Thus, the most typical algorithms used nowadays for self-driving vehicles are ones of the improvements A* or TangentBug-based algorithm. Moreover, these algorithms should consider the vehicle’s kinematic model.

As the paper discusses the possibility of developing a small, relatively cheap self-moving vehicle for nonintrusive object inspection, it is important to employ a not-high-performance computing device for initial data processing, fed data by a necessary set of sensors. Thus, one of the most suitable solutions is implementing the TangentBug algorithm family with its modifications for route planning. For safety radius computation, it is suggested to implement the InsertBug algorithm and its improvements, which take into account vehicle kinematics and sensor parameters.

## 5. Self-Driving Vehicles Positioning Principles and Navigation Control

### 5.1. General Principles of Global Positioning

One of the most critical questions in uncrewed-vehicle motion control is detecting its geographical position. Nowadays, most solutions use a Global Positioning System for this purpose, which implements satellite signal analysis to detect an object’s position.

The idea of satellite navigation takes its roots in the 1950s. When the first artificial Earth satellite was launched, American scientists led by Richard Kershner observed satellite signals and found that thanks to the Doppler effect, the frequency of the received signal rises when the satellite approaches and drops as it moves away. The essence of the discovery is that knowing the observer’s coordinates makes it possible to detect the satellite’s position and speed, and knowing the satellite’s placement makes it possible to see the observer’s speed and coordinates [72,73].

In 1973, the United States started the DNSS program, renamed later into NavStar, which aimed to launch satellites into medium Earth orbit, receive satellite signals using Earth-based equipment, and detect objects’ geographical coordinates with the use of specialized software. The program was renamed to its modern name, Global Positioning System (GPS), in December 1973.

With the spread of cellular communication, it became possible to produce various devices such as devices for geographic coordinate determination and data transmission devices used to transmit the coordinates of different transportation objects, such as cars, ships, aircraft, etc. These devices are called trackers. Gradually, with the development of microelectronics and software advances, as well as with the increase in cellular communication coverage, it became possible not only to transmit an object’s geographical coordinates but to perform other options, such as:

- Calculating the object’s location, speed, and movement direction based on the GPS satellites’ signals;
- Connecting external sensors to analog or digital inputs of a tracker;
- Reading data from the vehicle’s onboard equipment via either serial port or CAN interface;
- Storing a certain amount of data in the internal memory during communication breaks;
- Transferring received data to the central server for the following processing.

Received data can either be stored at a local storage device and transferred later to a centralized database or transmitted to the centralized database in real time via cellular communication channels. 

All modern tracking systems are identical. They have the same functionality, and the main differences are related to using components from different manufacturers and the functionality of particular techniques.

Therefore, for nonintrusive object inspection tasks with self-driving vehicles, the GPS module is responsible for detecting the current vehicle position and adjusting its motion algorithm if needed. The most typical implementation is in large areas, for example, at a gulf for maritime border control vehicles.

### 5.2. Software for Autonomous Moving Control

A vital component for any autonomous moving vehicle is software with built-in navigation maps and a report system. This software typically consists of two parts: the onboard vehicle’s software to control its motion, and remote monitoring software, to analyze and store tracking and other data. The onboard part is typically implemented using microcontrollers or single-board computers, while the remote monitoring part could be implemented either with the WEB-based software or as a desktop solution. 

The WEB service is typically used to control a small number of devices, while the main focus is operational GPS location monitoring. The WEB application is available from any computer independently of the operating system installed. This application typically has limited resources to control and analyze received data; thus, customized desktop solutions are used for more advanced and robust solutions. Furthermore, it should be considered that data in WEB-based applications typically are not aimed to be stored for a long time; as a rule, about one month. Thus, a report generated with such a system could be generated only for a limited period. With WEB-based applications, it is hardly possible to customize a program to specific needs and problems. Therefore, the undoubted advantage of WEB-based software is its ability to be started from any computer, tablet, or smartphone, neglecting the operating system installed. However, it is limited in the possibility of adjusting it to specific needs and the amount of data that could be processed. 

In the case of a special-purpose software system created, it could be tailored to specific task demands, including controlling the vehicle’s autonomous movements and analyzing data received from its additional sensors. In this case, all the data received from the vehicle are stored on remote storage, and they are constantly available and can be further processed with more powerful computers. Thus, it is possible to provide complex analyses on received data, including video image processing; build different analytical reports for any time; and work independently on Internet connections within local networks. 

Thus, for the aims of nonintrusive object inspection with the use of self-moving vehicles, the proper solution is the use of simple microcontroller-based software to control vehicle moving and send sensor data to the remote device, and custom advanced software within the powerful remote computer to store and analyze both vehicle’s movement and position data and additional nonintrusive inspection sensor data.

## 6. Maritime-Specific Sensors and Nonintrusive Object Inspection

Even though general principles of self-driving vehicles’ motion control are similar, maritime devices have their peculiarities. During an extended period, navigational safety control was not the priority task. All changed after 11 September 2001 [74]. Since then, different technologies and instruments have been implemented for maritime border control, especially underwater, to detect possible threats, such as military objects, divers, mines, smuggling, etc. Most of these technologies and instruments require the use of inspection equipment underwater, so underwater self-driving vehicles are in use to solve this task [75,76,77]. Although the general principles of route-planning algorithms are similar to terrestrial ones but with an additional degree of freedom, object recognition and nonintrusive inspection principles differ because of the different natures of water and air media. Thus, one of the most critical tasks here is object recognition, aimed at detecting nonconventionalities in common shapes or the sea floor and seeing the number of manmade objects compared to natural ones. Marine specifics make it not always possible to implement the same techniques as on-land inspection, which mainly relates to the different nature of aquatic media. Thus, the most popular methods for many years were those based on the use of acoustic waves for long-distance underwater information transmission [75]. These techniques employ different types of sound and sonar sensors [75,76,77]. They proved efficient in gathering underwater acoustic images and initially involved operators in recognizing suspicious objects. With the latest advances in digital technologies, different types of AI-based techniques are employed to make the recognition process automatic. 

Traditional methods typically used for underwater inspection use acoustic image analysis with image representation in grey-scale sonar or acoustic camera images and implement formal numerical analysis for object recognition [78]. A voting-based approach may be applied to enhance low-quality photos.

To enhance the quality of acoustic methods, additional analysis in the time–frequency domain proved efficient [75]. Such research provides faster and more reliable results than time-domain signal analysis, which allows for the detection and recognition of small targets in underwater control. 

The limitation of active sonar sensors, namely their relatively high cost, adverse side effects on marine wildlife, and easy visibility to intruders, led to the intensification of research on passive object detection methods [79,80,81,82].

Novel approaches implement AI elements to solve image recognition tasks [83,84]. The downside of underwater acoustic image analysis methods is the lack of datasets to train CNNs in the classification huge range of different object types, as most such techniques are developed to detect and classify military objects. In [85], the authors created massive datasets for typical underwater targets, such as drowning victims, airplanes, mines, wrecks, and seafloor images. They implemented an approach that involves semisynthetic data generation aimed at artificially creating additional photos to enlarge the dataset, followed by training the deep convolutional neural network with the whole dataset and fine-tuning it with 70% of the authentic images dataset, implementing the transferal of deep learning results obtained on semisynthetic data to natural images. 

In [86], a novel approach was proposed without using machine learning techniques to reliably detect underwater threats from sonar images. The method implements two steps: object area extraction and shape representation. Object area extraction represents image pixels as neural oscillators synchronizing with neighbors when their rhymes are similar. Shape representation detects conditions with the help of multiple resolution contours, which allows one to eliminate noises and—by implementing geometrical parameters—clearly detect object contours.

Another approach is grounded on underwater optical image processing. These techniques typically require cheaper equipment, and nowadays, pretty accurate solutions provide reliable underwater image analysis. However, in the early stages of underwater inspection, optical image-processing techniques were not that effective due to low-quality images taken [87]. Thus, additional strategies for image quality enhancement were applied. Traditional image-processing techniques assume grayscaled image processing in order to eliminate processing complexity and enhance object detection accuracy [88,89]. With the latest advances in video and image acquisition technologies, this group of methods became more popular due to cheaper hardware and the possibility to implement novel data-processing algorithms, providing reliable results even with low-quality optical images.

Even with advances in optical camera technologies, there is still an urgent need to enhance underwater images to improve the object recognition accuracy rate. In [90], a framework for underwater visual image recognition was proposed, which consists of three steps: a color correction algorithm, a super-resolution generative adversarial network, and an improved object recognition algorithm. The color correction algorithm allows one to eliminate unnecessary noise during image preprocessing. The proposed resolution improvement algorithm allows one to enhance image quality by deblurring and sharpening the image. A similar technique is widely used to enhance underwater image-processing quality [91]. Finally, the proposed object recognition algorithm allows one to improve object recognition accuracy. 

Another framework [92] proposes to process underwater optical camera images in the following three stages: in the first stage, an advanced median filter is used to eliminate noise from the image; in the second stage, a convolutional neural network is employed to image recognition with the samples from ImageNet—the world’s most extensive database for image recognition projects; in the third stage, preprocessed improved median filter images were used to tune a pretrained convolutional neural network (CNN) and classify objects in images. This approach demonstrated promising results in object recognition with small datasets used to train CNNs. 

Another framework [93] proposes to provide underwater image recognition based on deep learning and transfer learning methods. The idea is to label and train a deep learning neural network based on in-air optical image recognition for manmade objects, followed by moving acquired CNN knowledge to underwater detection of the same objects.

Threat detection accuracy could be increased with enhanced sensor capability, which typically leads to a significant increase in sensor price, and with the implementation of advanced signal processing approaches to multiple sensor signals [94].

Finally, genetic algorithms could be implemented to build a reliable maritime border inspection system. In [95], it was proposed to build system-of-system architecture (SoSA) for maritime border control, choosing all necessary components based on genetic algorithm-based model simulation. Such an approach allows one to select the SoSA optimal configuration depending on marine surveillance needs and constraints: low price, high precision, best area coverage, etc.

In [96], the authors developed a web-map visualization portal to track and receive data from remote self-driving autonomous underwater vehicles, which might be used for remote control and management of underwater vehicles. 

Another essential task in maritime border control with autonomous self-moving vehicles is the choice of self-driving motion control and route-planning algorithms [97]. Taking into account the specifics of the environment, it is not always possible to create an area map for vehicle orientation, and in most cases, there is a need to implement autonomous motion in an unknown environment. To solve this problem, a method that builds environment maps using underwater acoustic markers (also known as localization and mapping problems) is used [98]. 

In [99], a robust algorithm was proposed for underwater target recognition using optical camera images based on box partitioning. Such an approach overcomes problems related to noise or obstacles overlapping part of the image containing target points. However, this method, developed to be implemented for autonomous underwater vehicle docking, might be modified to detect different objects based on image processing.

Except for object recognition, a material-type detection also could be implemented in maritime border control. Thus, in [100], the authors use a neutron sensor for threat material analysis—laboratory experiments to distinguish rocks from explosives. 

Thus, the provided analysis showed two possible lines in underwater object inspection: the use of high-precision expensive acoustic sensors or high-resolution video cameras or creating cheaper multisensor systems with intelligent analysis algorithms to enhance aquatic control with the help of autonomous vehicles.

## 7. Nonintrusive Control with Self-Driving Vehicles

To implement nonintrusive control with the use of self-driving vehicles, a set of additional equipment might be installed. X-ray machines are one of the most used traditional techniques for nonintrusive object inspection. However, such a system is impossible to imagine in crowded places as a passive nondisturbing scanning system. Thus, other techniques should be implemented, such as odor recognition, spectrography methods, ultrasonic and ultraviolet signal analysis, etc., as described in [1]. 

## 8. Conclusions

In this paper, we have presented a review on modern advances in border control with the use of self-driving autonomous vehicles, starting from the typical structure of self-driving autonomous vehicles, analyzing specific sensors used for motion control, and typical algorithms used to implement autonomous movement according to preplanned route avoiding obstacles. Standard sensor types and motion control algorithms were reviewed and analyzed for their appropriate use in different tasks. Special attention was paid to maritime object inspection with the use of self-driving autonomous vehicles as one of the most promising fields for research with many open problems. The provided analysis showed the necessity of improving motion control algorithms and nonintrusive object inspection methods and equipment to increase accuracy and reliability and eliminate unnecessary disturbance to ordinary people while ensuring high security.

## Figures and Tables

**Table 1:** Comparison of the obstacle detection sensors.

Features	LIDAR	RADAR	Ultrasonic	Video Camera
Detect close objects	low	high	very high	low
Viewing angle	360°	360°	30°	~90°
Effective distance	~100 m	~0.15–250 m	0.03–10 m	~250 m
Operation in darkness	+	+	+	–
Speed detection	+	+	–	–
Device price	USD 70,000	~USD 200	1 USD/pcs	~USD 100
Processing unit price	USD 100+	USD 100+	USD 10	USD 100+

**Table 2:** Comparison of route-planning algorithms.

Algorithm	Computational Complexity	Guarantees Goal Achievement	Takes into Account Kinematics	Algorithm Speed	Works in Dynamically Changed Environment
Bug-1	Simple	Yes	No	Slow	Yes
Bug algorithms with the distance sensor	Medium	Yes	Yes/No	Medium	Yes
Tangent-bug	Medium	Yes	No	Medium	Yes
InsertBug	Medium	Yes	Yes	Medium	Yes
Potential field algorithms	Complex	No	No	Fast	No
Algorithms on graph	Complex	No	No	Fast	No
Hybrid search algorithms	Very Complex	Yes	Yes	Fast	Yes

## Data Availability

Not applicable.
